# Association between neuromyelitis optica and tuberculosis in a Chinese population

**DOI:** 10.1186/1471-2377-14-33

**Published:** 2014-02-20

**Authors:** Rui Li, Xiaonan Zhong, Wei Qiu, Aimin Wu, Yongqiang Dai, Zhengqi Lu, Xueqiang Hu

**Affiliations:** 1 Multiple Sclerosis Center, Department of Neurology, The Third Affiliated Hospital of Sun Yat-Sen University, No. 600 Tianhe Road, Guangzhou, Guangdong Province 510630, China

**Keywords:** Neuromyelitis optica, Tuberculosis, NMO-IgG

## Abstract

**Background:**

A number of reports have described the presence of tuberculosis (TB) in neuromyelitis optica (NMO) patients. However, a definite association between the two conditions has not been conclusively demonstrated.

**Methods:**

To investigate the association between NMO and TB in a Chinese population, we performed a retrospective review of hospital records of NMO patients, control patients and tuberculosis meningitis (TBM) patients from January 1, 1995 to December 31, 2011.

**Results:**

The frequency of preceding/simultaneous active pulmonary TB (PTB) was not significantly different between NMO patients (1.1%) and control groups (2.3% in myasthenia gravis, 1.1% in polymyositis or dermatomyositis, zero in idiopathic facial palsy and viral meningitis/meningoencephalitis). NMO cases differed from TBM cases in terms of demographics, course (recurrent or monophasic), cerebrospinal fluid analysis and magnetic resonance images. Two TBM patients shared partial clinical features with NMO (one of the TBM patients had a longitudinal extensive spinal cord lesion involving the holocord, and the other had optic neuritis before anti-tuberculosis treatment). NMO antibodies were only detected in NMO patients and not in TBM patients with myelitis or optic neuritis.

**Conclusions:**

We could not confirm previous suggestions of the association between PTB and NMO. Direct infection of the central nervous system by TB may mimic NMO in some respects, but whether NMO-like symptoms that develop during the course of TB should be considered and diagnosed as NMO is open to discussion.

## Background

Neuromyelitis optica (NMO) is a severe demyelinating disease of the central nervous system (CNS) that preferentially affects the optic nerve and spinal cord [1]. The disease is idiopathic and the discovery that patients are seropositive for aquaporin-4-specific autoantibody (AQP4-Ab or NMO-IgG) suggested the existence of an underlying immunopathogenic mechanism [1,2]. Although NMO usually occurs in isolation, reports over the past century have suggested an association between NMO and tuberculosis (TB) [3-8]. A study by Hughes and colleagues described three patients that developed acute necrotic myelopathy during the course of active pulmonary TB (PTB) [4]. A report from the Western Cape of South Africa described six patients with optic neuropathy and myelopathy with associated active PTB but no evidence of TB infection in the CNS [3]. Another study, also carried out in South Africa, suggested PTB-associated NMO developed in patients with active infection because 79% (11/14) of NMO patients had a preceding or simultaneous diagnosis of PTB [8]. Finally, Feng and colleagues described the benefits of anti-tuberculosis treatment in steroid-refractory Chinese NMO patients [9].

Although an association between NMO and TB has been suggested in a number of reports from different geographical regions, a definite association between the two conditions has not been conclusively demonstrated. Further investigation and clarification would be helpful in the diagnosis and treatment of patients who develop ‘NMO-like’ symptoms (longitudinal extensive myelitis and optic neuritis) during the course of TB. We therefore performed a retrospective study to investigate whether there is an association between NMO and TB in the Chinese population.

## Methods

### Ethics statement

Ethical approval was given by the medical ethics committee of the third affiliated hospital of Sun Yat-sen University with the following reference number: [2007]33. Written informed consent was obtained from the patient for publication of the clinical data and any accompanying images.

### Patients and selection criteria

To study the prevalence of PTB in NMO patients compared with controls, we performed a retrospective review of hospital records of patients with a diagnosis of NMO that were admitted to our hospital, The Third Affiliated Hospital of Sun Yat-Sen University, from January 1, 1995 to December 31, 2011. All NMO patients fulfilled the 2006 Wingerchuk criteria [10] (the 1999 Wingerchuk diagnostic criteria were used for several cases owing to a lack of NMO-IgG data [11]). Patients with evidence of sarcoidosis, vasculitis, clinically manifest systemic lupus erythematosus, Sjogren’s syndrome or another explanation for optic neuritis or myelitis were excluded [12]. The control groups consisted of patients with myasthenia gravis, polymyositis or dermatomyositis, idiopathic facial palsy, and viral meningitis/meningoencephalitis (VM). To avoid any confusion with aseptic meningitis/meningoencephalitis, patients were considered to have VM when either a viral etiology was confirmed or antiviral treatment was effective. VM was only diagnosed after ruling out bacterial, fungal, and non-infectious causes of meningitis (malignancy, autoimmune disorders, neurosarcoidosis) [13]. To ensure a similar background exposure to TB, control and NMO cases were matched for sex, age at time of admission (within 10 years), and date of admission (within a range of 5 years). If more than one matching control was identified per case, the one with the closest age at admission was included. These patients were used as controls because there is no known causal association with the risk factor under investigation (TB).

The following data were collected for all NMO and control cases: sex, age at onset of neurological symptoms, and the presence or absence of a diagnosis of active PTB [14]. Active PTB was defined either as a diagnosis of PTB preceding the onset of NMO by fewer than 6 months, or PTB that was not cured before admission for neurological disease. Duration from diagnosis of PTB to onset of neurological symptoms (if previously diagnosed with PTB), cerebrospinal fluid (CSF) data (if available), and NMO-IgG serum status (if available) was also collected for all cases.

To investigate whether patients with tuberculosis meningitis (TBM) shared partial clinical features to NMO patients fulfilling the 2006 Wingerchuk criteria, we also enrolled TBM patients admitted to our hospital during the same period using TBM criteria according to Thwaites [15]. Detailed data on clinical presentation, CSF reports, magnetic resonance imaging (MRI) reports, therapy, and outcomes were collected for NMO and TBM cases. Using MRI, spinal cord lesions extending over three or more vertebral segments on the spinal cord were defined as longitudinal extensive spinal cord lesions (LESCLs). Linear lesions were defined on T2-weighted imaging on MRI according to Misu et al. [16] as: (i) consecutive linear shape lesions on the sagittal plane and (ii) symmetric, with a preferential involvement in the central gray matter on the axial plane.

Serum NMO-IgG antibodies were tested according to the manufacturer’s instructions using aquaporin 4-transfected cells from a commercial sampling kit (EUROIMMUN AG, Luebeck, Germany).

### Statistical analysis

All statistical analyses were performed using Statistical Program for Social Sciences (SPSS) statistical software (version 16.0, San Francisco, CA, USA). *P* < 0.05 was considered statistically significant. Comparisons of the presence of PTB between NMO patients and controls were carried out using the Chi-squared test.

## Results

We enrolled 88 patients with NMO (67 diagnosed using the 2006 Wingerchuk diagnostic criteria and 21 using the 1999 Wingerchuk diagnostic criteria) and 352 controls into our study. The data on the presence of PTB in NMO and control groups are shown in Table 1. One NMO patient among the total 88 (1.1%) had a preceding diagnosis of PTB. The frequency of active PTB in NMO patients was not significantly different from that in patients with myasthenia gravis, polymyositis or dermatomyositis, facial neuritis, and VM (*P* > 0.05).

**Table 1:** Demography and presence of TB in NMO patients and controls

**Disease**	**Number of patients, n (M/F)**	**Age, mean ± SD, years**	**Presence of active PTB, n (%)**	***P *** **value**
NMO	88 (17/71)	34.1 ± 15.9	1 (1.1)	
Myasthenia gravis	88 (17/71)	33.5 ± 16.9	2 (2.3)	> 0.05
Polymyositis or dermatomyositis	88 (17/71)	35.6 ± 16.3	1 (1.1)	> 0.05
Idiopathic facial palsy	88 (17/71)	39.7 ± 16.0	0 (0)	> 0.05
VM	88 (17/71)	33.8 ± 14.5	0 (0)	> 0.05

*TB* tuberculosis; *PTB* pulmonary tuberculosis; *NMO* neuromyelitis optica; *VM* viral meningitis/meningoencephalitis; *M* male; *F* female; *P*: comparison for active *PTB* presence between *NMO* and each control groups.

**Figure 1:**
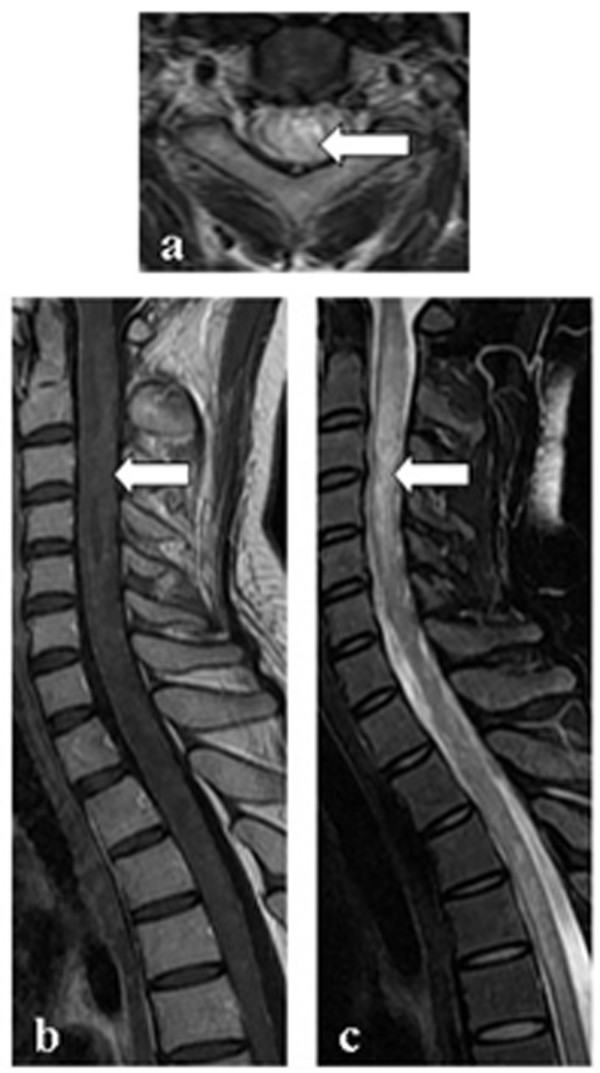
**Spinal MRI of the NMO patient with active PTB. a**: axial T2-weighted image showing a cervical lesion predominantly involving the central cord; **b**: T1-weighted contrast image revealing slight enhancement of the lesion; **c**: T2-weighted image showing a longitudinal extensive spinal cord lesion.

The single NMO patient who had a previous history of PTB showed no evidence (in either CSF or radiology reports) indicative of TB infection in the CNS. She was a 26-year-old woman who experienced visual blurring simultaneously in bilateral optic neuritis and hemiplegia of her left limbs, 2 months prior to admission. She was positive for NMO-IgG and the CSF protein concentration was 0.67 g/L, while glucose concentration and cell count were normal. Brain MRI showed non-specific white matter lesions. Spinal MRI showed a LESCL from C1 to T5 without evidence of meningeal enhancement or tuberculoma (Figure 1). The patient was diagnosed with NMO. She had consolidation in her chest X-ray 3 months prior to admission. During hospitalization she had a cough and her sputum was positive for acid-fast bacilli. Chest radiography revealed extensive bilateral confluent consolidation, predominantly involving the upper lobes. Ziehl-Neelsen microscopy showed no acid-fast bacilli in her CSF. After anti-tuberculosis treatment combined with methylprednisolone pulse therapy, her PTB recovered, but she had recurrent myelitis during the 17-month follow-up period. Special MRI procedures including diffusion and magnetic resonance spectroscopy were not performed because they were unavailable during that period. However, taking MRI features, discussion with neurosurgeons, and the later follow-up into consideration, intramedullary tuberculoma could be excluded.

**Figure 2:**
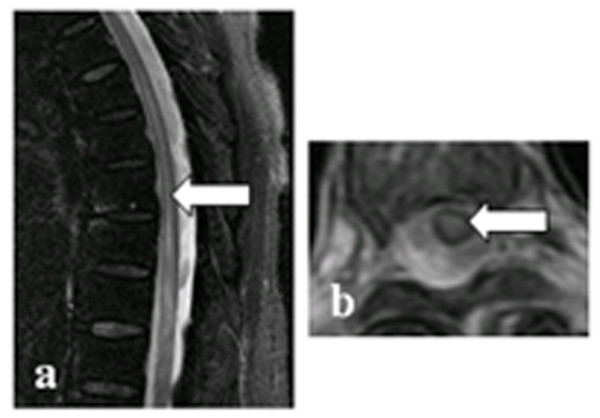
**Spinal cord lesion in an NMO patient.** The lesions are shown by arrows. **a**: T2-weighted image showing a linear-shape lesion; **b**: axial T2-weighted image showing a cervical lesion predominantly involving the central gray matter in the spinal cord.

**Figure 3:**
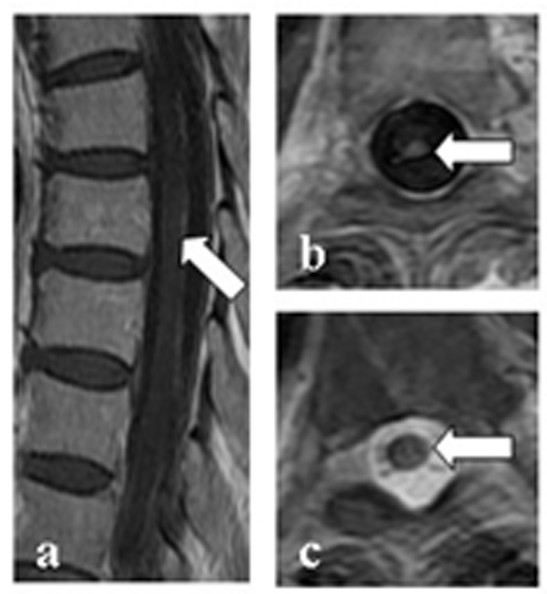
**Spinal MRI of a CNS-TB patient with spinal cord involvement. a**–**b**: meningeal and whole lesion enhancement on a T1-weighted contrast image; **c**: T2-weighted image showing an isolated lesion at the T5 level of the spinal cord.

**Figure 4:**
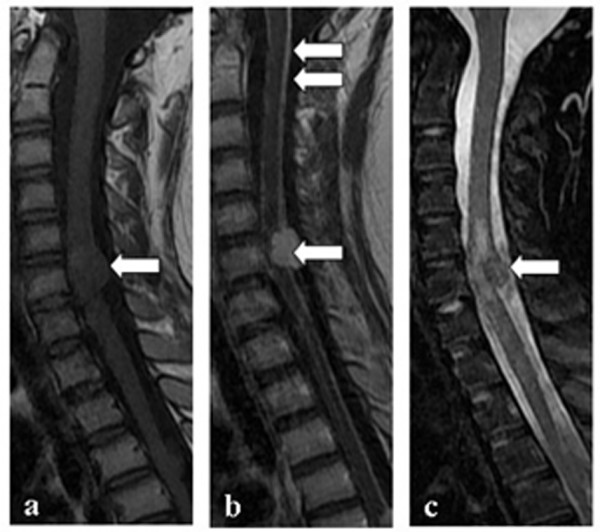
**Spinal MRI findings in a CNS-TB patient with spinal cord involvement. a**: tuberculoma with iso-intensity on sagittal T1-weighted image; **b**: T1-weighted contrast image showing enhancement of the whole lesion and meninges; **c**: tuberculoma with iso-intensity on a sagittal T2-weighted image.

**Figure 5:**
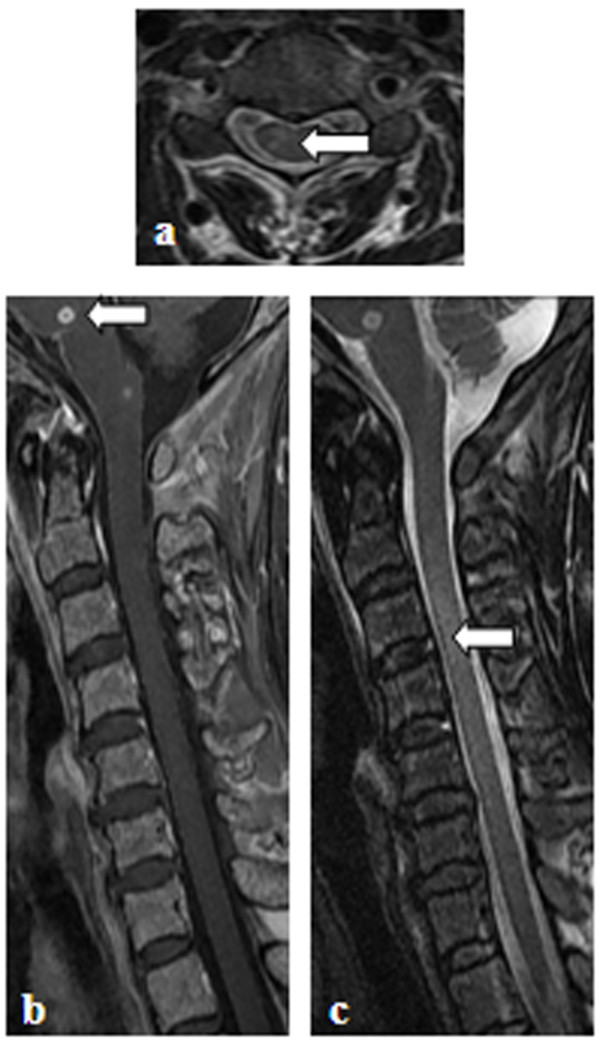
**Spinal MRI of a CNS-TB patient with spinal cord involvement. a**: axial T2-weighted image showing central gray matter involvement of the spinal cord. **b**: T1-weighted contrast image revealing tuberculoma enhancement in the medulla but no enhancement of the spinal cord lesion; **c**: T2-weighted image showing a longitudinal extensive spinal cord lesion without a well-demarcated boundary.

Table 2 shows the demographic and clinical features of NMO and TBM patients. Out of 92 TBM patients, 10 had myelitis (spinal meningitis, n = 2; tuberculoma, n = 1; meningitis combined with tuberculoma, n = 2; focal or extensive spinal lesions with hydrocephalus, meningitis or tuberculoma in brain MRI, n = 2; clinical manifestations indicating myelitis, n = 3). Two TBM patients had optic neuritis, of which one developed optic neuritis before anti-tuberculosis treatment. Compared with TBM related myelitis (TBM-MY), NMO patients had a higher female:male ratio (71:7 vs. 3:7), higher relapse rate of myelitis (75% vs. 0), higher seropositive rate of NMO-IgG (82.1% vs. 0%), and a higher frequency of normal cell count, protein, glucose and chloride concentration in the CSF. CSF TB-antibody positivity was found in 30% (3/10) of patients with TBM-MY, but not in NMO patients. Spinal MRI was performed in all NMO patients and 7/10 TBM-MY patients. There were no spinal MRI data for the three remaining TBM-MY patients because MRI was not available at the time. However, these patients presented with typical clinical symptoms indicating myelitis. Spinal cord lesions in NMO patients mostly extend over multiple cord levels involving central gray matter (linear lesions, shown in Figure 2) or holocord (LESCLs and spinal lesions with central/holocord involvement were found in 73.9% of patients). Compared with NMO patients, spinal image features of TBM-MY patients were different. Meningeal enhancement (57.1% of patients), or tuberculoma (42.9% of patients), which is characterized by oval lesions with low (or iso-) T1-weighted image signal, typical ‘target sign’ T2-weighted image signal, and nodular or rim enhancement in spinal cord was common (Figures 3 and 4). Although one TBM-MY patient had LESCLs, similar to the characteristic spinal MRI finding in NMO, the tuberculoma in his medulla suggested direct TB infection of the CNS (Figure 5). Methylprednisolone pulse therapy was administered to all NMO patients in the acute phase. During remission, six patients received additional immunosuppressants. All 92 TBM patients received anti-tuberculosis therapy (isoniazid, rifampicin, ethambutol plus pyrazinamide or streptomycin for 86 patients and isoniazid, rifampicin, ethambutol plus second-line drugs for the remaining six patients).

**Table 2:** Demographic and clinical features of NMO and TBM patients

	**NMO n = 88**	**TBM**		
		**Total n = 92**	**TBM-MY n = 10**	**TBM-ON n = 2**
Sex ratio, F/M	71/7	40/52	3/7	1/1
Age, mean ± SD, years	35.3 ± 14.4	33.5 ± 18.6	34.1 ± 13.9	19, 41
Relapse of myelitis, n(%)	66/88 (75.0%)	--	0/6 (0)	--
CSF				
Pleocytosis, n(%)	16/88 (18.2%)	92/92 (100.0%)	10/10 (100%)	2/2 (100%)
Increased protein level, n(%)	14/88 (15.9%)	92/92 (100.0%)	10/10 (100%)	2/2 (100%)
Decreased glucose level, n(%)	1/88 (1.1%)	92/92 (100.0%)	10/10 (100%)	2/2 (100%)
TB-antibody positivity, n(%)	0/45 (0)	27/92 (29.3%)	3/10 (30%)	0/2 (0)
* Mycobacterium tuberculosis*, n(%)	0/45 (0)	0/92 (0)	0/10 (0)	0/2 (0)
Seropositive NMO-IgG, n(%)	55/67 (82.1%)	0/5 (0)	0/3 (0)	0/1 (0)
Spinal MRI				
Linear lesions, n(%)	41/88 (46.6%)	--	0/7 (0)	--
LESCLs, n(%)	65/88 (73.9%)	--	1/7 (14.3%)	--
Central or holocord involvement, n(%)	65/88 (73.9%)	--	1/7 (14.3%)	--
Tuberculoma, n(%)	0/88 (0)	--	3/7 (42.9%)	--
Meningeal enhancement, n(%)	7/88 (8.0%)	--	4/7 (57.1%)	--
Follow-up duration, median (range), months	28.5 (12–106)	14.5 (5–96)	18.0 (6–84)	24, 17

*NMO* neuromyelitis optica; *TBM* tuberculosis meningitis; TBM-MY:TBM related-melitis; TBM-ON:TBM related-optic neuritis; *F* female; *M*: male; *CSF* cerebrospinal fluid; *MRI* magnetic resonance imaging; *LESCLs* longitudinal extensive spinal cord lesions.

## Discussion

Several reports have suggested PTB-related NMO is caused by an immune response to TB infection. Acute necrotic myelopathy or NMO occurred in a few patients with active PTB. A postmortem study found demyelination in the spinal cord without any evidence of TB involvement [3,4]. The study by Hughes and colleagues suggested tubercle bacillus, the bacterium that causes TB, shares antigens with myelin basic protein so that lymphocytes sensitized against mycobacterium recognize and attack myelin [4]. Recently, a report from South Africa described a higher proportion of active PTB in NMO patients compared with controls (79% vs. 14%, *P* = 0.0013 < 0.05) [8]. CNS-TB was excluded in all cases. In contrast to this previous report, we could not confirm an association of NMO with PTB. Only one (1.1%) of the 88 patients in the NMO group was previously diagnosed with PTB and this was similar to the percentage observed in the control group (*P* > 0.05). Western Cape Province in South Africa had an annual TB incidence of 517 per 100 000 in 2007 [8], while the estimated national incidence for China was considerably lower than that in South Africa (approximately 154 per 100 000 in 2006 according to World Health Organization reports [17], and even as low as 70 per 100 000 in some areas of China [18]). It is therefore likely that the prevalence of NMO in regions with a high incidence of TB is different from the idiopathic autoimmune form seen in areas of relatively low TB incidence.

Another possible explanation for TB-related NMO relates to a nonspecific adjuvant effect of TB that amplifies the immune response. It is relatively easy to induce an autoimmune disease in genetically susceptible animals via immunization with complete Freund’s adjuvant (containing inactivated *M. tuberculosis*) and an autoantigen [19-21]. Although we do not know to what extent the adjuvant effect of *M. tuberculosis* contributes to the pathogenesis of the experimental autoimmune encephalitis (EAE) model, a myelin-specific antigen in incomplete Freund’s adjuvant lacking *M. tuberculosis* can also induce EAE in marmosets [22], suggesting that, at least in some species, this agent is not necessary for induction of EAE.

It has also been proposed that some NMO cases are caused by a direct CNS infection with tuberculosis. In a small controlled study carried out by Feng and colleagues from China, positivity for *M. tuberculosis* DNA in the CSF of 2 out of 12 steroid-refractory NMO patients (confirmed using nested polymerase chain reaction and the long-term clinical efficacy of anti-tuberculosis treatment in these patients) suggested pathogenesis involves direct CNS infection [9]. However, it is possible that Feng et al.’s study may have been subjected to selection bias because the authors used the original criteria for NMO diagnosis (Wingerchuck 1999), which did not require NMO-IgG data, and their patients showed a high rate of CSF abnormalities (CSF protein levels were increased in 53.9% of patients and 50% had elevated CSF leukocyte counts) [9]. The present study used the newer, revised criteria that are more specific for NMO diagnosis than the original criteria [10]. Contrary to the findings in Feng et al.’s study, elevated protein level (15.9% of patients) and pleocytosis (18.2% of patients) in CSF were not common in our NMO patients (Table 2). Although these relatively low frequencies contrast with some reports from Western countries (which found abnormal CSF cell counts in the majority of patients in the acute phase [11,23]), our data are consistent with some previous studies carried out in Asia (27.3% of NMO patients had CSF pleocytosis in southern China [24], 25% in Japan [25], and 0 in Iran [26]).

Although our data show that TBM patients may develop myelitis or optic neuritis, many of these TBM patients can be differentiated from NMO patients on the basis of demographics, course (recurrent or monophasic), CSF, and neuroimaging features. However, it is worthwhile to note that two TBM patients shared partial clinical features with NMO; one of the TBM patients had LESCLs involving holocord, similar to the characteristic spinal MRI finding in NMO, and the other had optic neuritis before anti-tuberculosis treatment. This finding supports the notion that direct TB infection of the CNS may incidentally mimic NMO in some aspects. The presence of NMO-IgG has rarely been tested in ‘TB-related NMO’ cases in older studies because NMO-IgG was not discovered until 2004 [2]. Only one report has described two patients who developed NMO-like symptoms during the course of TB, but both patients showed seronegativity for NMO-IgG [7]. Consistent with this previous report, the two patients who shared partial clinical features with NMO in our study were also negative for NMO-IgG. We presume that these NMO-like symptoms with NMO-IgG negativity may differ from NMO with NMO-IgG positivity. In fact, reports have shown that NMO-IgG negative NMO differs clinically and epidemiologically from NMO-IgG positive disease and may even have a distinct pathogenesis [27]. Despite the high specificity of NMO-IgG for diagnosis of NMO [1,2,10], NMO-IgG negativity cannot rule out its diagnosis absolutely. This is because NMO-IgG levels and even serostatus can vary during follow-up [28]. Thus, diseases that mimic NMO, including CNS infections with a focus on the spinal cord and optic nerves, still have the potential to be diagnosed as NMO with NMO-IgG negativity. Recently, Miller and co-workers suggested that patients showing evidence of another explanation for ‘NMO-like’ symptoms should be excluded from NMO diagnosis [12]. Therefore, it is still debatable whether NMO-like symptoms that develop during the course of TB should be considered and diagnosed as NMO.

## Conclusions

The nature of NMO and its association with TB in the Chinese population may differ from populations from other regions. We could not confirm previous suggestions of an association between PTB and NMO. Direct TB infection of CNS may incidentally mimic NMO in some respects, but whether NMO-like symptoms that develop during the course of TB are diagnosed and treated as NMO is open to discussion. Although our findings argue against a causal association of NMO and TB, this study is limited by its retrospective design. We only focused on the relationship between NMO and active PTB and TBM because of limited datasets. A broader association of TB (for instance, distant or treated TB) and NMO should also be explored. Moreover, TB (the risk factor) does not occur at a sufficiently high frequency in either NMO patients or controls in our study to draw a definitive conclusion; therefore, a possible association between NMO and TB may be missed because of the small sample size. A larger prospective investigation with a long-term follow-up will be helpful in answering this question.

## Abbreviations

CNS: Central nervous system; CSF: Cerebrospinal fluid; LESCL: Longitudinal extensive spinal cord lesions; MRI: Magnetic resonance imaging; NMO: Neuromyelitis optica; PTB: Active pulmonary tuberculosis; TB: Tuberculosis; TBM: Tuberculosis meningitis; TBM-MY: Tuberculosis meningitis-related myelitis; VM: Viral meningitis/meningoencephalitis.

## Competing interests

The authors declare no conflicts of interest.

## Authors’ contribution

All authors have made substantial contributions to the intellectual content of the paper. RL and XZ contributed to the conception, design, and drafting of the manuscript. AW and YD contributed to the acquisition, analysis, and interpretation of data. WQ, ZL, and XH revised the manuscript critically for intellectual content. All authors read and approved the final manuscript.

## Pre-publication history

The pre-publication history for this paper can be accessed here:

http://www.biomedcentral.com/1471-2377/14/33/prepub
